# It takes a village: microbiota, parainflammation, paligenosis and bystander effects in colorectal cancer initiation

**DOI:** 10.1242/dmm.048793

**Published:** 2021-05-10

**Authors:** Xingmin Wang, Ram Babu Undi, Naushad Ali, Mark M. Huycke

**Affiliations:** 1 Nantong Institute of Genetics and Reproductive Medicine, Nantong Maternity and Child Healthcare Hospital, Nantong University, Nantong, Jiangsu 226018, China; 2 School of Medicine, Jiangsu University, Zhenjiang, Jiangsu 212013, China; 3 Department of Radiation Oncology, University of Oklahoma Health Sciences Center, Oklahoma City, OK 73104, USA; 4 Department of Internal Medicine, Section of Digestive Diseases and Nutrition, University of Oklahoma Health Sciences Center, Oklahoma City, OK 73104, USA; 5 Stephenson Cancer Center, University of Oklahoma Health Sciences Center, Oklahoma City, OK 73104, USA

**Keywords:** Colorectal neoplasms, Gut microbiome, Radiation-induced bystander effect, Neoplastic cell transformation, Carcinogenesis, DNA damage, Cancer stem cells, Cell-of-origin, Chromosomal instability, Mutation, Paligenosis, Bacteria, Cell dedifferentiation, Doublecortin-like kinase 1

## Abstract

Sporadic colorectal cancer (CRC) is a leading cause of worldwide cancer mortality. It arises from a complex milieu of host and environmental factors, including genetic and epigenetic changes in colon epithelial cells that undergo mutation, selection, clonal expansion, and transformation. The gut microbiota has recently gained increasing recognition as an additional important factor contributing to CRC. Several gut bacteria are known to initiate CRC in animal models and have been associated with human CRC. In this Review, we discuss the factors that contribute to CRC and the role of the gut microbiota, focusing on a recently described mechanism for cancer initiation, the so-called microbiota-induced bystander effect (MIBE). In this cancer mechanism, microbiota-driven parainflammation is believed to act as a source of endogenous mutation, epigenetic change and induced pluripotency, leading to the cancerous transformation of colon epithelial cells. This theory links the gut microbiota to key risk factors and common histologic features of sporadic CRC. MIBE is analogous to the well-characterized radiation-induced bystander effect. Both phenomena drive DNA damage, chromosomal instability, stress response signaling, altered gene expression, epigenetic modification and cellular proliferation in bystander cells. Myeloid-derived cells are important effectors in both phenomena. A better understanding of the interactions between the gut microbiota and mucosal immune effector cells that generate bystander effects can potentially identify triggers for parainflammation, and gain new insights into CRC prevention.

## Teaser

**Summary:** New mechanisms of sporadic colorectal cancer initiation link gut dysbiosis with recent concepts of parainflammation, paligenosis and bystander effects to drive chromosomal instability and malignant transformation in colon epithelial cells.

## Introduction

The human microbiome has gained wide recognition as an important contributor to human health and disease, with recent advances highlighting its role in cardiovascular, respiratory, autoimmune, psychiatric, metabolic, gastrointestinal and neurologic diseases (Lynch and Pedersen, 2016). Of equal importance is the microbiome's potential to contribute to carcinogenesis and to the global cancer burden (Rajagopala et al., 2017). Although well-defined mechanisms have been described for the initiation of cancer following infection with oncogenic pathogens, such as *Helicobacter pylori* and human papilloma viruses in gastric and cervical cancers, respectively, it was recently concluded that there is still no “direct evidence that the human commensal microbiome is a key determinant in the aetiopathogenesis of cancer” (Scott et al., 2019). Unfortunately, many gaps still exist in our knowledge regarding the underlying mechanisms of cancer initiation potentially caused by commensal microorganisms.

Sporadic colorectal cancer (CRC) is a leading cause of cancer and death due to cancer worldwide (Bray et al., 2018). As such, it is important to clarify the mechanisms of commensal microbiome-driven malignant transformation, not least because understanding such mechanisms could lead to new strategies preventing this disease. Sporadic CRC progresses in a well-defined manner during which normal colonic epithelium forms precursor lesions in the form of adenomas, which then become overtly malignant (Vogelstein et al., 2013). Genomic and epigenetic changes in these precursor adenomas can be readily investigated to identify those that initiate cellular transformation. In addition, numerous animal models of intestinal cancer have been developed, which provide additional insights into the role of commensals in carcinogenesis.

Human CRC is multifactorial in origin. Both host and environmental factors play major roles in influencing the genetic mutations and the transcriptional and epigenetic changes associated with this cancer (Lichtenstein et al., 2000) and, therefore, in the development of cells-of-origin (see Box 1, Glossary) (Visvader, 2011). Cofactors that affect sporadic CRC development include age, genetic background and lifestyle factors, e.g. diet, obesity, alcohol and smoking, together with extrinsic modifiers, such as non-steroidal anti-inflammatory agents (Song et al., 2020). The complex intertwining of these variables and factors in the etiology of CRC has rendered the identification of commensal-driven initiation mechanisms particularly challenging.

**Box 1. Glossary**

**Azoxymethane (AOM):** methyl-methylimino-oxidoazanium, a potent carcinogen and neurotoxin that induces colon cancer in rats and mice.

**Cancer stem cells (CSCs):** a small subpopulation of cells that can recapitulate a parent tumor through processes of self-renewal and differentiation.

**Cell-of-origin:** tumor-initiating cell that is a non-transformed precursor to cancer stem cells. They may arise from normal tissue-resident stem cells, such as those at the bottom of the intestinal crypt, or from fully differentiated post-mitotic cells. Cells-of-origin are non-malignant and cannot divide or reproduce indefinitely.

**Colibactin:** a non-protein bacterial compound synthesized by polyketide synthases and other enzymes, and encoded by a 54-kb genomic island designated *pks*. Colibactin is genotoxic and induces DNA double-strand breaks and chromosome aberrations in epithelial cells. It can induce colorectal carcinogenesis in animal models and has been associated with human CRC. The *pks* island is carried by selected strains of *Enterobacteriaceae*, including *Escherichia coli*.

**Crypt stem cell:** self-renewing cell at the base of crypts, which gives rise to differentiated cell types that maintain the integrity of the intestinal epithelium.

**Culture-enriched molecular profiling:** a combination of exhaustive bacterial culture techniques that use 16S rRNA gene sequencing to identify unique bacteria from complex ecological niches, such as the gastrointestinal tract.

**Dextran sulfate sodium (DSS):** a polysaccharide polymer that, when given orally, degrades the intestinal mucus barrier and induces severe colitis.

**Dysbiosis**: a perturbation in the healthy intestinal microbial population, which permits disease-associated pathobionts to emerge.

**Gnotobiotic:** an environment in which all present microorganisms are known; i.e. a gnotobiotic animal is an animal in which all present strains of bacteria and other microorganisms are known and defined.

**Inflammaging**: an inherent feature of the aging process characterized by the chronic, progressive and poorly controlled increase in proinflammatory status due to activation of innate immunity. Inflammaging is linked to numerous diseases of aging, including atherosclerosis, Alzheimer's disease, type II diabetes mellitus, osteoporosis and cancer among others.

**Interleukin 10 (IL10):** a potent anti-inflammatory cytokine that is produced by subsets of T cells and monocytes among many other cells that contributes to intestinal homeostasis and immune tolerance. Knockout of the *Il10* gene results in an unremarkable phenotype in mice unless colonized by pathobionts that induce colonic inflammation and microbiota-driven CRC.

**Lamina propria:** a thin vascular layer of connective tissue beneath the epithelium of a mucous membrane.

**Macrophage:** myeloid-derived immune cell found in virtually all tissues and especially enriched in the colon, which function as professional phagocytes to provide defense against exogenous pathogens, and/or to serve as sentinels for tissue homeostasis and tolerance to commensals. Macrophages have remarkable cellular plasticity and are readily polarized from a resting state (M0) to M1 or M2 phenotypes, depending on cues in their tissue microenvironment.

**Mucosal-associated invariant T (MAIT)** **cell:** unique major histocompatibility complex class I-related protein 1 (MR1)-restricted innate-like T cell that bridges innate and adaptive immunity.

**Mast cell:** myeloid-derived immune cell that is rich in histamine and heparin, expresses high-affinity receptors for IgE, and plays a key role in inflammation by releasing multiple mediators from storage granules into the tissue microenvironment.

**Mastocytosis:** a rare medical condition caused by excess numbers of mast cells congregating in tissues.

**Microbiota-induced bystander effect (MIBE):** a theory regarding colorectal carcinogenesis whereby gut bacteria polarize tissue macrophages to induce stress signaling and DNA damage in epithelial bystander cells, leading to cellular transformation and malignancy.

**Microbiota:** ecological communities of commensal, symbiotic and pathogenic microorganisms and viruses.

**Mono-association:** colonization with microbes of a single species.

**Neutrophils:** the most-abundant granulocytes, making up 40-70% of all white blood cells in humans. They are a crucial part of the innate immune system.

**Parainflammation:** an inflammatory state characterized by chronic activation of genes involved in innate immunity through persistent DNA damage.

**Pathobiont:** a commensal microorganism that causes disease when specific genetic, environmental or ecological conditions are altered.

**Prostaglandin-endoperoxide synthase 2** **(PTGS2**, also known as COX-2**):** an enzyme that converts arachidonic acid into prostaglandin H2, is expressed by macrophages during inflammation and plays a key role in colorectal carcinogenesis.

**Polymicrobial:** any condition involving multiple species of microorganisms.

**Reactive oxygen species (ROS)**: chemically reactive molecules that, among others, include superoxide, hydroxyl radical and peroxides. ROS are by-products of aerobic metabolism and can play important roles in cell signaling. They can also lead to cellular damage of proteins, lipids and nucleic acids.

**Radiation-induced bystander effect (RIBE):** compilation of phenomena in which irradiated cells can induce stress signaling and DNA damage in bystander cells through diffusible mediators or gap-junction communication.

**T cell:** a type of lymphocyte; they are divided into several subsets, each of which has a crucial role in adaptive immune responses. Among others, there are T_H_1, T_H_2, T_H_9, T_H_17, cytotoxic, Treg, Tr1 and T_CM_ cells.

**Tr1 cell:** T regulatory type 1 cell that is distinct from T_H_1, T_H_2 and T_H_17 cells. Tr1 cells express an anergic phenotype that induces high-level production of the anti-inflammatory cytokine IL10 but little to no IL2, IL4 or IFNG.

**Treg:** regulatory T cell; a subset of T helper cells (CD4^+^) that express the FOXP3 transcription factor and CD25, and help maintain tolerance by suppressing or downregulating the induction and proliferation of effector T cells.

This Review focuses on microbiota-driven mechanisms that initiate sporadic CRC and help elucidate the disease's known cofactors. The microbiota comprises living members of the microbiome – bacteria, fungi, protozoa and other eukaryotes, and is often also considered to include viruses and bacteriophages (Box 1). The preponderance of research on microbiota-driven mechanisms for cancer initiation in CRC involve bacteria, although a few studies have highlighted potential roles for viruses, bacteriophages and fungi (Coker et al., 2019; Goel et al., 2006; Hannigan et al., 2018). Here, we focus on bacterial studies and models that have helped to elucidate causal relationships among the gut microbiota, dysbiosis (Box 1) and CRC. Emphasis is placed on bystander effects that lead to mutations, epigenetic reprogramming and the cellular transformation of colon epithelial cells. We draw parallels between a theory for the microbiota-induced bystander effect (MIBE; Box 1) and the well-characterized radiation-induced bystander effect (RIBE; Boxes 1 and 2) (Burdak-Rothkamm and Rothkamm, 2018). Bacterial traits that promote the growth of malignant cells, facilitate epithelial-mesenchymal transition and metastasis, modulate cancer therapy or function as probiotics in CRC prevention are not discussed, as these topics have been recently reviewed elsewhere (Allen and Sears, 2019; Eslami et al., 2019; Janney et al., 2020; Tilg et al., 2018).

**Box 2. Radiation*-*induced bystander effect (RIBE)**

RIBE generates numerous bioreactive molecules that – with considerable implications for cancer initiation – chronically induce stress signaling and DNA damage in bystander cells. In addition to the direct effects on irradiated cells, ionizing radiation causes a range of cellular responses, particularly DNA damage and CIN (Bach et al., 2019; Hoevenaar et al., 2020) in unirradiated bystander cells exposed to irradiated cells (Mothersill and Seymour, 2004). RIBE was initially observed in individuals who had undergone whole-body irradiation and were found to have chromosome-breaking factors (clastogens) in their blood many years later (Goh and Sumner, 1968), indicating persistent clastogen production (Marozik et al., 2007; Pant and Kamada, 1977). This effect has been confirmed using irradiated tissue culture cells (Nagasawa and Little, 1992) and in bone marrow transplants form irradiated rodents (Watson et al., 2000). Hematopoietic and mesenchymal cells, including macrophages, are common effectors of RIBE (Burr et al., 2010; Dong et al., 2015; Lorimore et al., 2008; Pampfer and Streffer, 1989).

RIBE activates multiple signaling pathways in bystander cells, including NF-κB, MAPK and JNK (Azzam et al., 2002), leading to altered expression of stress response genes (e.g. *PTGS2* and *NOS2*), activation of DNA damage repair (p53/p21 and ATM/ATR), epigenetic modification (through miRNA and/or CpG island methylator phenotypes), and proliferation, apoptosis and death of bystander cells (Azzam et al., 2002; Ilnytskyy et al., 2009; Najafi et al., 2014; Sokolov and Neumann, 2018; Zhou et al., 2005).

Bystander effects occur through diffusible mediators and gap-junction intercellular communication (Azzam et al., 2003). Mediators include ROS and mutagenic products of lipid peroxidation (Emerit et al., 1996; Lorimore et al., 2008; Wang et al., 2012), TNF-α, IL1A, and IL8 (Dong et al., 2015; Fu et al., 2016; Yang et al., 2012), extracellular vesicles (Al-Mayah et al., 2015; Ariyoshi et al., 2019; Mo et al., 2018; Rastogi et al., 2018; Szatmári et al., 2019), TGF-β1 (Gow et al., 2010); cell-free DNA (Konkova et al., 2019; Sergeeva et al., 2017), and cysteine protease cathepsin B and insulin-like growth factor receptor (Peng et al., 2017). Irradiation can also release chromatin particles that are aberrantly integrated into the DNA of bystander cells, leading to genomic damage (Kirolikar et al., 2018), and can induce signaling in bystander cells through pattern recognition receptors (Schaue et al., 2015).

## Gut microbiota and dysbiosis in CRC

The gut microbiome is a complex ecosystem of living microorganisms that contains mixtures of metabolites, structural elements and other bioactive molecules (Berg et al., 2020). The colon contains a high density and diversity of microbiota (∼10^11^ microorganisms per ml) (Sender et al., 2016). By comparison, the human distal small intestine has a much lower density of bacteria than the colon (∼10^8^ microorganisms per ml). Despite a 94% larger surface area (Helander and Fandriks, 2014), the incidence of adenocarcinomas in the small intestine is 99% less compared with that in the colorectum (Qubaiah et al., 2010). The magnitude of this difference, and the many animal models of intestinal cancer showing a requirement of microbiota for cancer initiation, provide compelling evidence that the colorectal microbiota play a key role in colon carcinogenesis. Several mechanisms have been described for how commensals might generate the genomic damage and epigenetic changes that precede malignant transformation in colon epithelial cells. These mechanisms are considered in this review, albeit with a focus on MIBE.

Numerous murine models have provided useful insights into microbiota-associated CRC initiation and progression. These models can be placed into several different categories: (1) carcinogen induced (see Box 3 carcinogen-based models of CRC); (2) engineered mutations in CRC driver genes or in genes that modulate driver genes; (3) initiated by agents or mutations that disrupt the intestinal barrier, leading to inflammation and; (4) gene knockouts that lead to intestinal inflammation (see Table 1). The role of the microbiota in these models is often investigated by using antibiotic treatment or is carried out under conditions that are either gnotobiotic, i.e. free of germs, mono-associated and defined as being polymicrobial, or free of specific pathogens (Box 1 and Table 1). As expected, each model has its advantages and disadvantages. For example, gnotobiotic models that investigate specific microbiota in intestinal carcinogenesis are inherently biased by systematic alterations in murine immune responses that arise from gnotobiosis and, potentially, affect inflammation-associated mechanisms (Kennedy et al., 2018). Chronic antibiotic therapy to induce intestinal dysbiosis might also not mimic the dysbiosis that precedes human sporadic CRC. Mutations in driver genes can oversimplify the complex mutational landscape and the epigenetic changes that occur in oncogenes and tumor suppressors in sporadic CRC. Engineered or spontaneous mutations in *Apc*, as exemplified in the mouse *Apc*^Min/+^ model of intestinal tumorigenesis (Su et al., 1992), do not accurately recapitulate the pathology of human sporadic CRC. Additionally, mutations in *Apc* primarily generate adenomas in the rodent small intestine, whereas precursor lesions in humans occur in the large intestine (McIntyre et al., 2015). Overall, although these models can help to refine and confirm microbiota-associated mechanisms in CRC, no model has as yet faithfully recapitulated the spectrum of genetic, epigenetic and histological findings observed in human sporadic CRC.

**Box 3. Carcinogen-based models of CRC**

Azoxymethane (AOM) is commonly used to induce colon tumors in rodent models by intraperitoneal injection. AOM is a metabolite of 1,2-dimetylhydrazine and a metabolic precursor to methylazoxymethanol. The metabolic activation of these alkylating agents leads to the aberrant methylation of guanine at the *O^6^*- and *N^7^*-positions (Rogers and Pegg, 1977). When AOM treatment is followed by oral administration of DSS, an agent that disrupts the mucus and the intestinal barrier that protect against colonic inflammation, the incidence of colon tumors increases together with their size and frequency (Tanaka et al., 2003). The resulting adenomas show characteristics common to precursor lesions in human CRC, such as β-catenin activation and induction of *PTGS2*. An early study using gnotobiotic rats found that colon cancer initiation by 1,2-dimethylhydraine depended on the microbial conversion of 1,2-dimethylhydrazine to AOM (Reddy et al., 1975). This report was among the first to implicate the intestinal microbiome in CRC carcinogenesis. Although the AOM/DSS model is still widely used, it relies on an exogenous carcinogen and, thus, does not recapitulate the potential mechanisms for carcinogenesis involving endogenous mutagenesis.

**Table 1.**
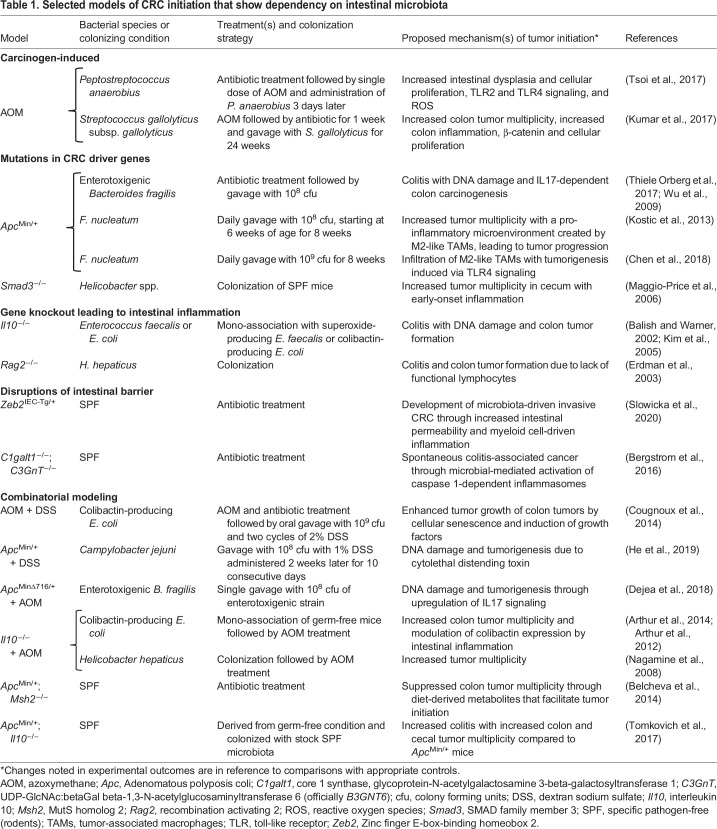
Selected models of CRC initiation that show dependency on intestinal microbiota

In addition to animal models, culture-enriched molecular profiling provides further evidence for the involvement of microbiota in human CRC (Box 1) (Lau et al., 2016), as do metagenomic sequencing (Ranjan et al., 2016) and metabolomics (Brown et al., 2016). These technologies allow for the comprehensive investigation of the colon microbiota and its relationship to CRC. Metagenomic investigations in particular have identified associations between CRC and imbalances in the intestinal microbiota, such as those associated with dysbiosis (Wirbel et al., 2019). In a recent study of fecal samples and tumor biopsies, bacterial phylotypes were correlated with human colon adenomas and sporadic CRC (Wirbel et al., 2019). Data from large CRC cohorts have identified multiple bacterial species and phylotypes that are associated with CRC, such as *Bacteroides fragilis*, *Fusobacterium* spp., *Porphyromonas* spp., *Parvimonas micra*, *Prevotella* spp., *Alistipes finegoldii*, *Gemella morbillorum* and *Thermanaerovibrio acidaminovorans* among others (Dai et al., 2018; Wirbel et al., 2019). Additional findings suggest that other pathobionts (Box 1), such as *Salmonella enterica* (Lu et al., 2016; Mughini-Gras et al., 2018) and *Streptococcus gallolyticus* subsp. *gallolyticus* (Kumar et al., 2017), together with viruses (Hannigan et al., 2018) and fungi (Coker et al., 2019), are also linked to colorectal carcinogenesis. Unfortunately, these strategies are unable to identify or to address the specific mechanisms by which commensals or pathobionts generate the genomic damage and the epigenetic changes that lead to the malignant transformation of colon epithelial cells.

Several bacterial phenotypes have been shown to initiate CRC. The phenotypes and their underlying mechanisms include toxin-induced damage to colon epithelial cell DNA as caused by colibactin-producing *Escherichia coli* (Box 1), alterations in cell signaling pathways that regulate genomic stability as caused by enterotoxigenic *B. fragilis* and the polarization of tissue macrophages (Box 1) or the generation of lipid peroxidation and reactive oxygen species (ROS; Box 1) as caused by *Enterococcus faecalis* and *Fusobacterium nucleatum* (Allen and Sears, 2019; Faїs et al., 2018; Wang et al., 2012). A summary of proposed CRC-initiating mechanisms produced by these specific pathobionts is provided in Table 2. As discussed below, commensal-driven immune mechanisms represent a common theme for many of the proposed mechanisms for CRC initiation and provide an integrated perspective on the origins of cellular transformation (Irrazabal et al., 2014; Janney et al., 2020; Lasry et al., 2016). However, these mechanisms are best understood in the context of what is currently known about the genetics, cells-of-origin (Box 1), precursor lesions, histological morphology and inflammatory milieu of CRC. These topics are, therefore, reviewed below.

**Table 2.**
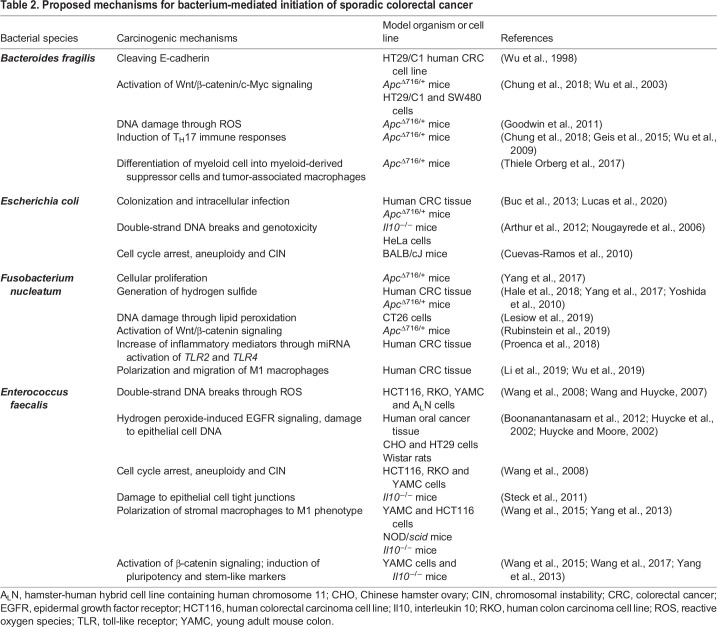
Proposed mechanisms for bacterium-mediated initiation of sporadic colorectal cancer

## Colorectal cancer origins and genetics

Our current understanding of the origin of most cancers, including sporadic CRC, postulates that they arise as a consequence of genetic and/or epigenetic changes in somatic cells. These changes may be inherited or result from environmental insults to the genome, such as those discussed above. Over time, cells undergo selection and clonal expansion to culminate in cancer (Hanahan and Weinberg, 2011; Vogelstein et al., 2013). The transcriptomic profiling of CRCs has identified four consensus subtypes of tumor cells based on changes in gene expression: microsatellite instability immune, canonical, metabolic and mesenchymal (Guinney et al., 2015). However, this approach has been challenged because it does not define the cells-of-origin used to develop these transcriptional subtypes (Dunne et al., 2016). Recently, RNA sequencing (RNA-seq) analysis of single cells isolated from CRC tumors has identified transcriptional groups that correspond to canonical and metabolic consensus subtypes (Lee et al., 2020). Despite this progress, these approaches have yet to define the mechanisms of CRC initiation. By contrast, the analysis of somatic mutation signatures of human sporadic CRC may be better able to help clarify the potential variety of initiating mechanisms. For example, whole genome sequencing of 60 CRC biopsies identified 14 distinct single-base substitutions in >50% of tumors from 49 biologically relevant signatures that had been curated from 2650 human cancers (Alexandrov et al., 2020). Nine CRC signatures (64%) were associated with well-defined mutational or epigenetic processes, e.g. deamination of 5-methylcytosine, defective DNA-mismatch repair and ROS. Moreover, ROS-related mechanisms have been implicated in CRC initiation for enterotoxin-producing *B. fragilis*, *F. nucleatum* and superoxide-producing *E. faecalis* (Table 2). In an elegant study using colibactin-producing *E. coli* infection of clonal human colon organoids, a specific mutational signature was identified and, subsequently, found in 5–7.5% of CRC biopsies (Pleguezuelos-Manzano et al., 2020). Of particular interest was evidence for this signature in healthy human colon crypts, suggesting that *E. coli*-driven DNA damage is an initiating event in a small percentage of CRC cases. However, mechanisms underlying many of the other common mutation signatures have yet to be identified.

### Precursor lesions in colorectal cancer

Colon polyps are precursor lesions for CRC and occur in 20-53% of adults in the USA aged >50 years (Strum, 2016). These lesions are classified as adenomas (75%) or serrated lesions (25%), with the latter consisting of hyperplastic polyps, sessile serrated lesions and traditional serrated lesions (Crockett and Nagtegaal, 2019; Strum, 2016). Unlike large (≥10 mm) polyps, diminutive (1-5 mm) and small (6-9 mm) polyps often regress (Strum, 2016). Aberrant crypt foci are an additional type of precursor lesion that are small and difficult to detect. These foci can be induced by colon-specific carcinogens in animal models, exhibit hyperplasia or dysplasia, and harbor the typical genetic and epigenetic changes found in adenomas and serrated lesions (Lopez-Ceron and Pellise, 2012). Azoxymethane (AOM; Box 1) is the prototypical carcinogen used in these models and requires activation by the gut microbiota to induce mutations (Table 1). Approximately 85% of CRCs arise from conventional adenomas and, following malignant transformation, show an average of 66 nonsynonymous mutations (Vogelstein et al., 2013). The source of these mutations is largely unknown, although enterotoxigenic *B. fragilis*, colibactin-producing *E. coli*, *F. nucleatum* and superoxide-producing *E. faecalis* have all been shown to damage epithelial cell DNA (Tables 1 and 2).

The most common genomic alternation in adenomas is chromosomal instability (CIN) (Shih et al., 2001b), which is characterized by extrachromosomal DNA, as well as insertions, deletions, amplifications and translocations in existing chromosomes. CIN can occur very early in precursor lesions and leads to the overexpression of oncogenes, and to the silencing of tumor suppressors to drive cellular proliferation and malignant transformation (Bach et al., 2019; Hoevenaar et al., 2020; Nowak et al., 2002). CIN and microsatellite instability are genomic hallmarks of most precursor lesions. Not surprisingly, a subset of CRCs express both microsatellite instability and CIN (Bolhaqueiro et al., 2019). These genomic changes are dynamic, accumulate over time and can lead to malignant transformation (Blokzijl et al., 2016; Tomasetti et al., 2013). Among the gut microbiota, *E. faecalis* in particular can generate CIN and aneuploidy in epithelial cells through ROS and MIBE (Table 2).

Sporadic CRCs arising from serrated lesions characteristically feature mutations in *KRAS* or *BRAF* (Crockett and Nagtegaal, 2019). *BRAF* mutations result in the widespread methylation of regions that comprise a high frequency of cytosine followed by guanine, i.e. CpG islands, causing the silencing of tumor suppressors. For example, hypermethylation of the *MLH1* promoter region, which is involved in DNA mismatch repair, results in microsatellite instability, with typically >700 mutations in malignant tumor cells. *Fusobacterium* species and *E. faecalis* are associated with human CRC that expresses the CpG island methylator phenotype (Lennard et al., 2016; Tahara et al., 2014). It remains to be determined whether these associations are causal or, more simply, due to microsatellite instability tumors that exhibit enhanced colonization of bacteria.

### Transforming somatic cells into cancer stem cells

CRC emerges from colon epithelial cells with mutations and epigenetic changes in genes that drive transformation, e.g. *APC*, *TP53* and *MLH1* as tumor suppressors, and *KRAS* and *CTNNB1* as oncogenes (Vogelstein et al., 2013; Yaeger et al., 2018). Most cancer cells in a tumor mass contain the original genomic changes that led to malignancy, although tumor genomes also continuously adapt to changing tumor microenvironments. A minority of cells within a tumor mass (1-2%) are cancer (or tumor) stem cells (CSCs), which are characterized by self-renewal and lineage-production (Box 1). These cells can recapitulate the original tumor mass upon seeding to an appropriate niche (Zeuner et al., 2014). Lineage tracing in genetically engineered CRC models shows that CSCs can arise from self-renewing crypt stem cells (Box 1) that are exposed to transformation-promoting processes, e.g. chemical or radiation injury or chronic inflammation. Thus, these cells are candidates for the cells-of-origin for CRC (Barker et al., 2009). However, experimental evidence also suggests that constitutive activation of Wnt signaling or engineered mutations of *Apc* and *Kras* in fully differentiated colon epithelial cells can also induce transformation and form CSCs (Schwitalla et al., 2013; Tetteh et al., 2016).

Recent studies have shown that cells-of-origin for CRC can also arise from somatic cells that are fully (or terminally) differentiated and undergo reprogramming. This process is likely to occur following chronic mucosal injury or inflammation. The orchestration of tissue repair and regeneration involves a sequence of cellular autodegradation, metaplastic gene expression, reactivation of mammalian target of rapamycin complex 1 (mTORC1), and cell cycle re-entry through a process termed paligenosis (Boxes 1 and 4) (Burclaff and Mills, 2018; Willet et al., 2018). Somatic cell dedifferentiation and proliferation during paligenosis occurs via crosstalk among mTORC1 via Hippo-YAP, Notch1 and Wnt/β-catenin pathways (Kaur and Moreau, 2019). The process of paligenosis has been observed in gastric and pancreatic tissues (Willet et al., 2018). Ongoing injury, as might occur during chronic inflammation, and checkpoint failure following DNA damage could lead to cellular proliferation, repetitive cycles of paligenosis, and to the accumulation of mutations in a ‘cyclical hit’ model of carcinogenesis (Burclaff and Mills, 2018; Miao et al., 2020b). This way, cells may become fixed in a state of dedifferentiation or induced pluripotency and may propagate mutations as cells-of-origin for CSCs. Massively parallel transcriptome analyses of individual CRC tumor cells have shown that tumor cells co-segregate with normal stem-like and proliferative cell types (Lee et al., 2020), providing support for this concept, although direct evidence is still needed for these processes in colorectal carcinogenesis. To the extent that dysbiosis in the gut microbiota induces chronic mucosal injury and parainflammation (Box 1), bacteria might be expected to contribute to paligenosis and the eventual creation of cells-of-origin for CSCs.

**Box 4. Paligenosis and a ‘cyclical hit’ model for carcinogenesis**

Paligenosis is the mechanism by which fully differentiated cells can re-enter the cell cycle in response to tissue injury and DNA damage (Willet et al., 2018). This is an evolutionarily conserved repair process that begins with the quenching of the mammalian target of rapamycin complex 1 (mTORC1), a master regulator of cellular energetics. Inhibition of mTORC1 occurs through injury-activated DNA damage-induced transcript 4 (DDIT4), which helps to ‘license’ post-mitotic cells to re-enter the cell cycle (Miao et al., 2020b). This is followed by increased autophagocytic activity to remove differentiated cellular features and damaged organelles. The reactivation of mTORC1 is associated with increased levels of injury-induced interferon-related developmental regulator 1 (IFRD1), thereby deactivating a key p53 checkpoint, and enabling cells to progress into and through the cell cycle (Miao et al., 2020a). This process occurs in response to tissue injury and does not occur in normally dividing stem cells, although a role for mTORC1 in stem cell activity has yet to be defined. Disruptions in paligenosis have been associated with tumorigenesis in the stomach and pancreas but not the colon (Miao et al., 2020b; Willet et al., 2018). Bypass or loss of function of the IFRD1/p53 checkpoint should allow for the ‘unlicensed’ division of cells, with the propagation of DNA mutations in a ‘cyclical hit’ model of carcinogenesis (Burclaff and Mills, 2018).

Researchers have modeled murine intestinal tumors that originate from fully differentiated colon epithelial cells that had undergone reprogramming and dedifferentiation. For example, fully differentiated intestinal epithelial cells in mice that are engineered to constitutively activate nuclear factor kappa-light-chain-enhancer of activated B cells (NF-κB) and Wnt/β-catenin undergo dedifferentiation and malignant transformation (Schwitalla et al., 2013). Enterotoxigenic *B. fragilis*, *F. nucleatum* and *E. faecalis* can each activate Wnt/βcatenin signaling in epithelial cells (Table 2). Similarly, tuft cells that express doublecortin-like kinase 1 (*Dclk1*) can serve as cells-of-origin for intestinal tumors in *Apc* knockout mice exposed to colitis-inducing agents (Westphalen et al., 2014). Colon tuft cells are fully differentiated, long-lived intestinal epithelial cells located in the crypt base and are scattered along the crypt axis. These cells perform chemosensory functions, modulate stromal immunity and regulate epithelial DNA damage responses through paracrine mechanisms (Middelhoff et al., 2017). Although *Dclk1* expression was noticed in tumors initiated through MIBE by using polarized macrophages infected with *E. faecalis* (Wang et al., 2015), the role of this gene in paligenosis and cancer initiation remains unclear. Intestinal tumors can also be generated in mice by selectively engineering mutations in *Apc* and *Kras* in post-mitotic cells expressing carbonic anhydrase as a differentiation marker (Tetteh et al., 2016). In summary, it appears that – in murine models – almost any cell type within a colon crypt is capable of being transformed into cells-of-origin for CRC. How cells-of-origin divide to form early precursor lesions in sporadic CRC will provide significant additional insight regarding their source and is discussed in the following section.

### Top-down versus bottom-up morphogenesis in colorectal adenomas

**Fig. 1.**
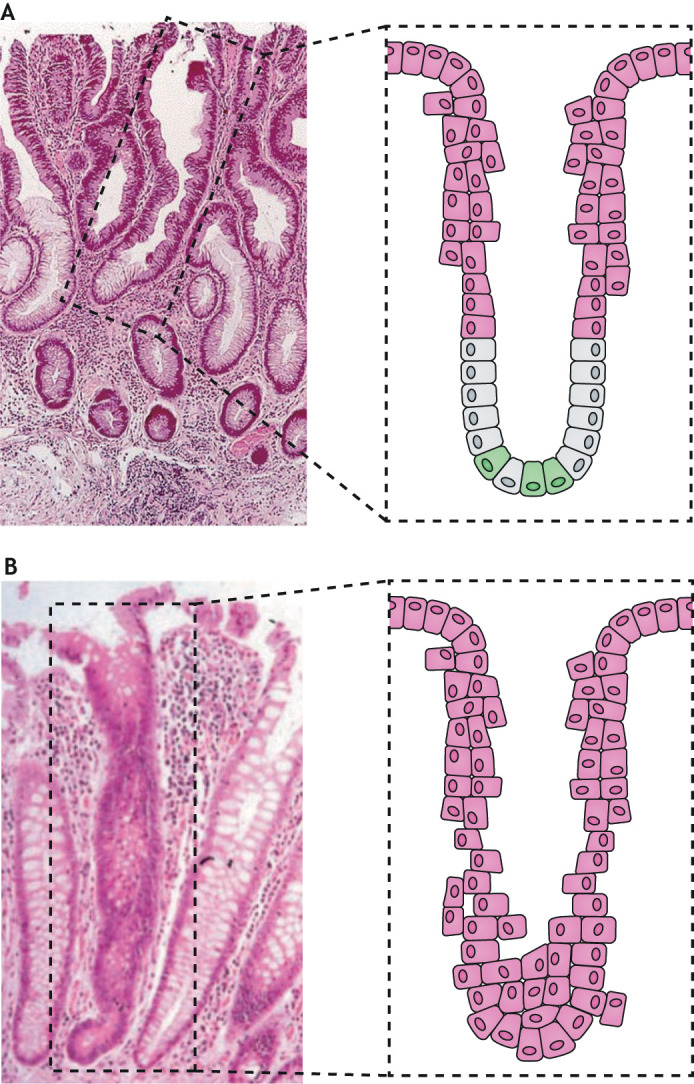
**Early features of colon crypt morphogenesis in human adenomas.** (A) Top-down morphogenesis. H&E-stained section of a typical small adenomatous polyp in the human colon. The highlighted structure shows dysplastic epithelium at the top of a crypt that is contiguous with the histologically normal epithelium at the base of the crypt. Notice the abrupt transition between dysplastic cells (pink) and the normal-appearing crypt stem cells and differentiating epithelial cells near the crypt mid-point (green and grey). The photomicrograph was reproduced with permission from Shih et al. (2001a). (B) Bottom-up morphogenesis. H&E-stained colonic section from an individual with familial adenomatous polyposis showing a monocryptal adenoma, in which all cells are dysplastic (pink). Dysplastic stem cells that are located at the crypt base always drive the dysplastic morphology for an entire crypt. The photomicrograph was reproduced with permission from (Preston et al., 2003).

The careful morphological analysis of diminutive colon adenomas has helped clarify the location of epithelial cells-of-origin in sporadic CRC (Cole and McKalen, 1963; Maskens, 1979; Shih et al., 2001a). CSCs have commonly been thought to arise from crypt stem cells. If this were true, then transformed cells within small tumors, as noted by Shih et al., “…should give rise to new, completely dysplastic crypts that branch as lesions expand. Histopathological examination, however, has long shown that this expected pattern is not observed…” (Shih et al., 2001a). These investigators confirmed that adenomas in the human colon undergo a pattern of ‘top-down’ morphogenesis with cells located in the upper portion of colon crypts and in zones between crypt orifices rather than at the crypt base – as would be expected if crypt stem cells were the cells-of-origin (Fig. 1A). Nearly all small, sporadic human colon adenomas show top-down morphogenesis, with dysplastic cells at the crypt surface that show active Wnt/β-catenin signaling and loss of *APC* (Shih et al., 2001a). Epithelial cells at the crypt base that appear histologically normal do not show Wnt signaling and loss of *APC*, two features considered to be characteristic of CRC. A top-down pattern of morphogenesis had been difficult to reconcile with the concept of CRC arising from normal crypt stem cells. However, recent studies have helped with this by demonstrating that fully differentiated colon epithelial cells can be reprogrammed into cells that can serve as cells-of-origin for CSCs (Adachi and Scholer, 2012; Burclaff and Mills, 2018; Greten, 2017). These findings have reframed concepts about the origin of top-down morphogenesis.

Top-down morphogenesis may also partly explain why crypt stem cell markers have not proven useful for identifying the cells-of-origin in human sporadic CRC. A retrospective analysis of 11 putative markers in colon adenomas found that only four had higher levels of expression when compared to normal epithelial cells (Jang et al., 2016). However, no consistent pattern of marker expression was observed in this study.

Support for top-down morphogenesis was found in several murine models of intestinal cancer (Schwitalla et al., 2013; Tetteh et al., 2016; Westphalen et al., 2014). Tuft cells are fully differentiated somatic cells – making them prime candidates for cells-of-origin for CSCs. These cells constitutively express Dclk1, a protein kinase with tumor promoter activity and, as with crypt stem cells, persist in colon crypts for long periods (Chandrakesan et al., 2017; Ong et al., 2014; Westphalen et al., 2014). Such persistence can allow for the gradual accumulation of mutational and epigenetic burdens that drive transformation (Westphalen et al., 2014). In mice, expression of *Dclk1* has been identified in cells-of-origin for CSCs (Chandrakesan et al., 2014, 2017; Nakanishi et al., 2013; Westphalen et al., 2014). In *Apc*^Min/+^ mice, Dclk1 correlates with enhanced pluripotency and increased self-renewal (Chandrakesan et al., 2017). Finally, inactivation of *Dclk1* in *Apc* mutant mice markedly suppresses adenoma multiplicity (Chandrakesan et al., 2014).

Murine models of CRC also provide evidence for ‘bottom-up’ adenoma morphology. This particular histology has been observed in intestinal adenomas that arise after activation of the Wnt/β-catenin signaling pathway in rapidly dividing crypt stem cells (Barker et al., 2009; Zhu et al., 2009). Similarly, and depending on context, colon tumors have been shown to also originate from non-cycling reserve stem cells in the crypt base (Powell et al., 2012). Unicryptal microadenomas, i.e. dysplastic epithelia cells that occupy an entire crypt, represent bottom-up morphogenesis and are observed in polyps from patients with germline *APC* mutations (Fig. 1B) (Preston et al., 2003). Similarly, bottom-up morphogenesis is found in *Apc*^Min/+^ mice. Finally, activating mutations in *Kras* or NF-κB that drive dedifferentiation in cells with *Apc* mutations produce intestinal lesions in mice with both top-down and bottom-up morphology (Cammareri et al., 2017).

Overall, there is strong evidence from murine models that the cells-of-origin for CSCs in CRC can arise from fully differentiated somatic cells. Although migration of crypt stem cells from their normal location at the base to the upper crypt and to intrazonal regions between crypts cannot be entirely excluded as an explanation for top-down morphology, a more parsimonious explanation would be that post-mitotic epithelial cells dedifferentiate to become cells-of-origin. One potential driving force for such a process would be tissue injury from chronic parainflammation driven by dysbiosis and pathobionts. Multiple rodent models of intestinal cancer show that the gut microbiome can degrade or breach intestinal barriers to induce inflammation and initiate CRC (Tables 1 and 2). CSCs are inherent to CRC formation, although cells-of-origin for CSCs have yet to be identified in bacterium-driven models. Finally, it must be noticed that no murine model of CRC, particularly those due to germline mutations, has been shown to recapitulate the top-down morphogenesis seen in sporadic human adenomas. Therefore, new models or new analyses of established models are needed, which can generate cells-of-origin through commensal-driven mutations and reprogramming, and create top-down morphogenesis.

### Immune cells and parainflammation in colorectal cancer initiation

Innate and adaptive immune systems, along with disruptions to intestinal barriers, such as mucus, tight junctions and basement membrane, play pivotal roles in CRC initiation (Lasry et al., 2016). The importance of inflammation to the development and progression of precursor lesions was highlighted in large human clinical trials showing that treatment with aspirin or non-steroidal anti-inflammatory drugs can reduce the risk of adenomas and CRC by 20-40% relative to placebo (Katona and Weiss, 2020). Colon adenomas are often infiltrated by polarized macrophages, neutrophils, mast cells and/or activated T cells, including regulatory T cells (Tregs) (see Box 1; McLean et al., 2011). Observations suggest that most colon adenomas exist in an intermediate state, somewhere between overt chronic inflammation, similar to that seen in colitis and the basal tolerance of the normal intestinal mucosa that shows no evidence of immune activation. This state has been termed parainflammation (Lasry et al., 2016; Pribluda et al., 2013).

There is scant evidence concerning the role of dysbiosis in parainflammation within human colon adenomas. Selected members of the gut microbiota have been shown to activate T cells that contribute colorectal carcinogenesis. This is best exemplified by enterotoxigenic *B. fragilis*-induced colitis and tumorigenesis in *Apc*^Min/+^ mice, which both require activation of T helper type 17 (T_H_17) cells via signal transducer and activator of transcription-3 (Stat3) (Wu et al., 2009). In this model, *B. fragilis*-induced expansion of Tregs limits the availability of interleukin 2 (IL2) and allows for T_H_17 responses to develop and drive carcinogenesis (Geis et al., 2015). Innate lymphoid cells and IL22 have also been shown to promote colorectal cancer in a *H. hepaticus*-driven model of carcinogenesis (Kirchberger et al., 2013). In *Apc*^Δ468/+^ mice, the dysregulation of Tregs induces mastocytosis (Box 1) and drives the adenoma-carcinoma sequence (Gounaris et al., 2009). Recent work has shown that mucosal associated invariant T (MAIT) cells (Box 1) are also linked to cancer initiation, growth and metastasis (Yan et al., 2020). MAIT cells are clusters of unconventional T cells primarily located in the lamina propria of the intestine (Box 1), lung and female genital tract. MAIT cell development requires the presence of both the intestinal microbiota and B cells (Treiner et al., 2003). The selection and expansion of MAIT cells is regulated by the major histocompatibility complex related protein 1 (MR1). MR1 recognizes a narrow range of unstable pyrimidine intermediates that are derived from bacteria and fungi (Corbett et al., 2014). MAIT cells occur in increased numbers in human CRC (Zabijak et al., 2015) but, as yet, there is no consensus concerning their role in anti- or pro-tumor responses. In a model of lung cancer, mice that lack the *MR1* locus or in which MR1 is blocked by antibody show decreased tumor growth and reduced metastasis. These effects can be reversed by MAIT cell transplantation (Yan et al., 2020). The modulation of MAIT cells, other T-cell subsets and innate lymphoid cells by the gut microbiota requires further investigation to clarify their roles in colorectal carcinogenesis.

Histological analyses of human and murine intestinal adenomas, and of *E. faecalis*-triggered CRC, have shown mast cells infiltrating these lesions (Gounaris et al., 2009; Yang et al., 2013). In these lesions, mast cells and macrophages are associated with inorganic polyphosphates that activate neutrophils to release extracellular ‘traps’ composed of DNA. These traps have been implicated in tumor progression (Arelaki et al., 2018). Colon cancer cells can also attract mast cells that promote proliferation and tumor growth by releasing soluble mediators (Yu et al., 2018). The nature of these mediators and their overall contribution to cancer initiation, however, remains unclear.

Macrophages perform many niche-specific functions in the healthy colon (Davies et al., 2013). Many studies focus on their role in the cancer microenvironment as tumor-associated macrophages (TAMs) (Vitale et al., 2019). These cells, however, should be considered separately from the polarized macrophages that are involved in CRC initiation. TAMs are heterogeneous and express pro-inflammatory M1 macrophages that are positive for CD68, CD80, inducible nitric oxide synthase (iNOS) and TNF-α, or anti-inflammatory/pro-tumoral M2 macrophages that are positive for CD163, CD206 and Arg1 markers, or both – depending upon type, stage and immune composition of a tumor (Sainz et al., 2016). TAMs enhance the growth of existing tumors, promote metastasis, contribute to resistance to chemotherapy, and generally confer a poor prognosis. TAM plasticity is defined as the ability to polarize or depolarize, or adapt their phenotype, depending on signals in the microenvironment. For example, Toll-like receptor agonism, interferon-γ (IFNG) and TNF-α favor an M1 phenotype, whereas IL4, IL10 (Box 1), IL13, and TGF-β induce macrophage polarization from M1 to M2 (van Dalen et al., 2018). Perturbation of the gut microbiota can promote tumor growth and invasion through modulation of macrophage plasticity. The complexity of these interactions had been suggested for human sporadic CRCs, wherein increased *Firmicutes* and *Bacteroides* were observed in the gut microbiota of patients showing high infiltrations of M1 TAMs in their tumor masses (Kikuchi et al., 2020). In a xenograft model, intestinal antibiotic-induced dysbiosis activated (or polarized) macrophages and increased the growth of a grafted human CRC cell line (Wan et al., 2018).

Although there is extensive literature describing the functions of TAMs, much less is known about the role of macrophages and colonic parainflammation in tumor initiation. Histologic analyses have shown that an abundance of polarized macrophages is present in most colon adenomas (McLean et al., 2011). As the lesions they infiltrate are not malignant, these cells are not TAMs, usually express an M1 phenotype and show activation of NF-κB, p38 mitogen activated-protein kinase (MAPK) and c-Jun N-terminal kinase (JNK) pathways (Hardwick et al., 2001). In addition, prostaglandin-endoperoxide synthase 2 (PTGS2, also known as COX-2) (Box 1), TNF-α and iNOS are often induced (Ambs et al., 1998; Chapple et al., 2000). So far, mechanisms that maintain a state of chronic macrophage polarization and parainflammation have not been well defined. Among the numerous genes induced in M1-polarized macrophages, the one encoding the pro-carcinogenic enzyme PTGS2 (Box 1) plays an outsized role in cancer initiation. Inhibition of PTGS2 decreases tumor multiplicity in numerous murine models and, according to many randomized controlled clinical trials, prevents CRC (Fischer et al., 2011; Janakiram and Rao, 2014; Katona and Weiss, 2020). Among pathobionts, *in vitro* inhibition of PTGS2 by drugs or through gene knockdown in *E. faecalis*-infected macrophages diminished the development of CIN in bystander epithelial cells (Wang and Huycke, 2007). However, in *Apc*^Min/+^ mice, specific deletion of *PTGS2* in myeloid cells but not in intestinal epithelial cells, showed no effect on intestinal tumor multiplicity (Cherukuri et al., 2014), yet again highlighting the difficulties that can occur when extrapolating findings from animal models to a human disease.

Agents that deplete colon macrophages and, thus, block or eliminate PTGS2 and other inflammatory cytokines, protect against CRC. In the *E. faecalis*-triggered IL10 knockout model of CRC, liposome-encapsulated clodronate selectively reduced colon macrophages, and prevented colitis and CRC (Watanabe et al., 2003; Yang et al., 2013). Clodronate is a bisphosphonate that depletes tissue macrophages by selectively inducing apoptosis. Zoledronate and alendronate are closely related bisphosphonates that were recently shown to suppress azoxymethane-induced CRC in rats (see Box 3) (Madka et al., 2020). Meta-analyses of bisphosphonate therapy in humans, which is usually given to treat osteoporosis, show that it is associated with marked reductions in CRC risk (Bonovas et al., 2013; Thosani et al., 2013). Overall, these data highlight the importance of parainflammation in CRC initiation and its possible associations with M1-polarized macrophages, Tregs, Tr1 (Box 1) and T_H_17 cells. A more-holistic theory for CRC initiation is discussed in the following section that links dysbiosis and the gut microbiota with parainflammation, paligenosis and cells-of-origin for CSCs.

## Microbiota-induced bystander effect and initiation of colorectal cancer

MIBE is a term that describes the paracrine interaction of polarized macrophages with colon epithelial cells to generate DNA damage and to initiate malignant transformation (Wang et al., 2017). Among several mechanisms by which intestinal pathobionts initiate CRC (Table 2), MIBE uniquely links immune activation and parainflammation to genomic instability, epigenetic change, the development of cells-of-origin through induced pluripotency and top-down morphogenesis. Inflammation is a recognized hallmark of cancer (Hanahan and Weinberg, 2011) and colonic parainflammation is likely to play a prominent role in the initiation of sporadic CRC.

**Fig. 2.**
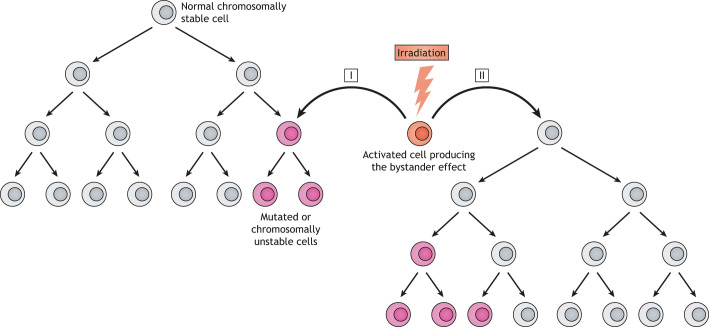
**Radiation-induced bystander effect (RIBE).** The bystander effect can be generated by cells (orange) that are directly ‘activated’ by irradiation or can arise from the progeny of irradiated cells (not shown). Myeloid and mesenchymal cells are the cells that typically generate a bystander effect. Subsets of normal chromosomally stable bystander (or target) cells (gray) that are exposed to diffusible mediators generated by irradiated cells develop genomic instability (pink cells). DNA damage is usually observed in either the first generation of exposed cells (I) or in the descendants of exposed cells (II). Modified with permission from Springer Nature (Lorimore et al., 2003).

Our group has studied MIBE as a generalized mechanism for the transformation of somatic epithelial cells into CSCs (Wang et al., 2013; Wang and Huycke, 2007, 2015; Yang et al., 2013). We have investigated MIBE by using an *E. faecalis* colonized IL10 knockout mouse model for colitis-associated CRC. In this model, *E. faecalis*, as a human commensal and opportunistic pathobiont, chronically polarizes colon macrophages to a pro-inflammatory M1 phenotype that generates endogenous mutagens and inflammatory cytokines. Colon epithelial cells targeted by M1 macrophages sustain double-strand DNA damage, mutations, tetraploidy and aneuploidy (Wang et al., 2008; Wang and Huycke, 2007; Yang et al., 2012). Repetitive exposure of primary colon epithelial cells to macrophages polarized by *E. faecalis* leads to malignant transformation (Wang et al., 2015). As bystanders to these macrophages, intestinal epithelial cells develop Wnt/β-catenin signaling and induce transcription factors associated with induced pluripotency (Wang et al., 2017). Conceptually, MIBE is analogous to well-characterized observations in radiation biology, showing that irradiated cells can produce soluble factors that generate mutations and genomic instability in non-irradiated bystander cells (Mothersill and Seymour, 2004). A comparison of MIBE to radiation-induced bystander effect (RIBE) has provided important new insights into the mechanisms of CRC initiation (see Box 2 and Fig. 2).

**Fig. 3.**
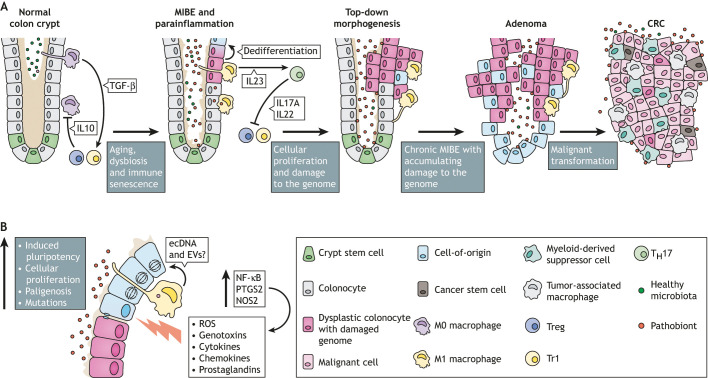
**Proposed mechanism for sporadic colorectal cancer (CRC) initiation by a microbiota-induced bystander effect (MIBE).** (A) *Normal colon crypt*: Normal homeostasis in a colon crypt involves healthy microbiota, adequate mucus and tight junctions to form an intact intestinal barrier, and efficient shedding and engulfment of apoptotic cells at the crypt apex. Tissue macrophages are maintained in a tolerogenic state (M0) through the regulatory influences of TGF-β expressed by phagocytic macrophages and anti-inflammatory actions of IL10 produced by Tr1 and Treg cells. *MIBE and parainflammation*: Aging leads to increased immune senescence and dysbiosis, which impair the homeostatic inhibition of mucosal macrophages. Disruption of the intestinal barrier, e.g. through the loss of mucus or tight junction integrity, contributes to aberrant macrophage polarization. This results in parainflammation and the polarization of tolerogenic M0 macrophages to pro-inflammatory M1 macrophages. Parainflammation is maintained through T_H_17 pathways. *Top-down morphogenesis*: Prolonged MIBE-mediated changes lead to abnormal, i.e. dysplastic, cells that collect in the upper part of the crypt. Although dysplastic colonocytes accumulate DNA and epigenetic changes, most small adenomas revert to normal histology upon restoration of the intestinal barrier or resolution of dysbiosis. *Adenoma*: However, some proliferative lesions continue to develop during chronic parainflammation. At this stage, precursor lesions acquire sufficient genomic instability to become cells-of-origin that can be transformed into cancer stem cells (CSCs). *CRC*: Chronic MIBE, therefore, may culminate in the emergence of CSCs, and tumors that feature colonizing pathobionts and tumor-associated immune cells, such as tumor-associated macrophages and myeloid-derived suppressor cells. (B) Close-up of a colon crypt affected by dysbiosis, loss of barrier integrity, MIBE and parainflammation. These processes activate NF-κB signaling in M1 macrophages, which induces PTGS2 and iNOS proteins, and the generation of genotoxic and pro-proliferative paracrine factors, such as reactive oxygen species (ROS), genotoxins (e.g. 4-HNE), cytokines (e.g. TNF-α), chemokines (e.g. CCL2) and prostaglandins. Additional mediators of the bystander effect may include extracellular DNA (ecDNA) and extracellular vesicles (EVs). These diffusible factors cause epithelial cell injury, leading to induced pluripotency and the expression of stem-like markers (dedifferentiation), as well as proliferation through paligenosis, mutations, genomic instability, epigenetic modifications, the activation of β-catenin and, ultimately to the creation of cells-of-origin for CSCs.

In the MIBE paradigm, the activation of immune effector cells by intestinal commensals creates endogenous mutational stress on colon epithelial cells, while simultaneously instigating epigenetic changes that drive malignant transformation (Fig. 3). MIBE shares many characteristics with RIBE. Macrophages in MIBE are chronically polarized and serve as effectors for DNA damage and CIN. This is analogous to the polarization of macrophages by irradiation to cause RIBE (Burr et al., 2010). In these scenarios, a bystander effect is induced by polarized macrophages, generating diffusible mediators and cytokines that damage DNA in targeted cells. Examples of these mediators include 4-hydroxy-2-nonenal (4-HNE), a breakdown product of ω-6 polyunsaturated acids and by-product of PTGS2, TNF-α and ROS (Burr et al., 2010; Yan et al., 2006; Yang et al., 2012; Zhou et al., 2005). In addition, both MIBE and RIBE depend on PTGS2 activity (Wang et al., 2013; Zhou et al., 2005). In one animal model, colon macrophages were implicated as the primary effector cells for MIBE in CRC (Yang et al., 2013), although neutrophils and Tregs are also likely to play important roles (Lakritz et al., 2015; Rao et al., 2006).

### Mouse models of MIBE

MIBE has been primarily investigated using *E. faecalis* as a pathobiont in the colitis-associated *Il10* knockout (*Il10^−/−^*) murine model of CRC (Kim et al., 2005; Wang et al., 2008, 2015, 2012; Wang and Huycke, 2007; Yang et al., 2013, 2012). Under germ-free or pathogen-free conditions, *Il10^−/−^* mice are healthy and show no evidence of colitis or cancer. However, when these mice are colonized with selected pathobionts, such as *E. faecalis*, *E. coli* or *Helicobacter* spp., colon macrophages become chronically polarized, followed by the subsequent formation of cancer (Balish and Warner, 2002; Kim et al., 2005; Sellon et al., 1998). These macrophages bear resemblance to the M1-polarized macrophages observed in human colon adenomas. These models, however, are complicated by observations that inflammation alone does not drive carcinogenesis. Colonization with *E. faecalis* causes colitis in *Il10*^−/−^ mice but the extracellular production of superoxide appears necessary for cancer formation (Wang et al., 2012). Similarly, *E. coli* causes colitis in *Il10*^−/−^ mice but colibactin is required for colon tumors to form (Arthur et al., 2012). Such findings highlight the importance of specific bacterial phenotypes, e.g. the production of colibactin or the formation of superoxide – and not just the global presence of species or genera, in the initiation of microbiota-driven carcinogenesis.

The *Rag2*^−/−^/*Apc*^Min/+^ model of intestinal carcinogenesis represents a variant of the bystander effect following intestinal colonization with *H. hepaticus* or *Campylobacter jejuni* (Rao et al., 2007). Instead of promoting adenoma formation, these bacteria initiate distant tumors in the mammary tissue of *Rag2*^−/−^/*Apc*^Min/+^ mice. Similar observations have been made using Tg(C3-1-TAg)cJeg/JegJ mice colonized with *H. hepaticus* (Lakritz et al., 2015). In these models, inflammatory cytokines (e.g. TNF-α and IFNG), together with ROS and diffusible mutagens, are implicated as mediators for the bystander effect.

### MIBE and CRC risk factors

Any credible theory regarding initiation of CRC should conform to known risk factors for CRC. MIBE largely fits this requirement. Age is by far the greatest risk factor for CRC. In the USA, the age-adjusted incidence rate for CRC per 100,000 increases from ∼10 for individuals of 40-49 years of age to >140 for individuals of >80 years of age (Ansa et al., 2018). This increased risk is not solely due to the occurrence of spontaneous somatic mutations in adult crypt stem cells. For colon crypt stem cells, the average rate of spontaneous mutation was estimated to be 40 per year, with small intestinal crypt stem cells exhibiting a similar rate (Blokzijl et al., 2016). However, the overall age-adjusted incidence of CRC is 100-fold higher than it is for adenocarcinoma of the small intestine (Qubaiah et al., 2010). It would, therefore, seem that age-related mutations alone do not account for the higher incidence of CRC.

Lifestyle is an additional major risk factor for CRC and, as such, has been extensively investigated. Unfortunately, collinearity between dietary factors, such as sugar, fruit, vegetable and fiber intake, and other behaviors or traits, such as physical activity, alcohol consumption, smoking and body-mass index, limit the ability to isolate their independent effects on the overall risk of developing CRC (Chan and Giovannucci, 2010). In addition, the magnitude of risk due to these factors is considerably smaller than age, with average age-adjusted relative risks typically >80-95% less.

Dysbiosis in gut microbiota has become the latest risk consideration for CRC (Janney et al., 2020). Evidence for this largely derives from associations between intestinal dysbiosis − or loss of healthy intestinal commensals that limit the outgrowth of pathobionts – and CRC (Dai et al., 2018; Levy et al., 2017; Sobhani et al., 2011; Wirbel et al., 2019). Perturbations in the gut microbiota correlate with changes in diet, antibiotic use and systemic or localized inflammation, and become increasingly prevalent with advancing age (Buford, 2017). Dysbiosis allows CRC-associated pathobionts to emerge and, potentially, to initiate CRC; for example, through mechanisms as listed in Table 2. Furthermore, as people age, the immune system becomes increasingly subjected to immunosenescence and to chronic, low-grade inflammation or ‘inflammaging’ (Box 1) (Franceschi et al., 2000; Fulop et al., 2017). This effect has been demonstrated in mice, where age-associated dysbiosis of the gut microbiota promotes intestinal permeability and systemic inflammation mediated by TNF-α (Thevaranjan et al., 2017). The dual effects of dysbiosis and immunosenescence/inflammaging is likely to cause dysregulation of host tolerance to gut commensals and a predisposition to the aberrant activation of innate immune cells that generate MIBE. Immune activation is seen as parainflammation in the precursor lesions for sporadic CRC.

### Macrophages as primary effectors for MIBE

Macrophages serve several key roles in gut homeostasis (Joeris et al., 2017). They clear apoptotic cells via efferocytosis and transiting bacteria via phagocytosis, perform tissue remodeling, and interact with T cells and innate lymphoid cells. They sample luminal antigens and transfer those antigens to neighboring dendritic cells. Under normal conditions, intestinal macrophages resist polarization from a resting M0 phenotype. This is primarily due to the anti-inflammatory effects of IL10 produced by Tregs and Tr1 cells (Bogdan et al., 1991; Chen et al., 2003; Vieira et al., 2004), TGF-β produced by phagocytic cells (Perruche et al., 2008), and lack of expression of innate response receptors (Smythies et al., 2005). Despite homeostatic tolerance, human intestinal macrophages retain robust phagocytic and bactericidal activity.

However, with advancing age, macrophages develop pro-inflammatory phenotypes, particularly in adipose and hepatic tissues (Jackaman et al., 2017). Peritoneal macrophages from aged mice are hyper-responsive to activation and show enhanced production of ROS (Smallwood et al., 2011). Other studies, however, found that aged peritoneal and splenic macrophages show decreased responsiveness to inflammatory stimuli (Yoon et al., 2004) but, a careful evaluation of age-related changes in human or rodent intestinal macrophages has yet to be carried out. Despite a lack of evidence, the importance of macrophage polarization to tumor initiation is supported by studies that used *Il10*^−/−^ mice colonized with *E. faecalis* or *E. coli*, transgenic mice overexpressing the transcription inhibitor Zeb2 or the *Apc*^Min/+^ mouse model, as well as the treatment of mice with AOM and dextran sulfate sodium (DSS; Box 1) to induce colitis and tumor formation. In these systems, depletion of colon macrophages or blockage of macrophage migration in response to C-C motif chemokine ligand 2 (CCL2) effectively inhibits microbiota-driven intestinal inflammation and reduces or abolishes tumor formation (Gu et al., 2019; Popivanova et al., 2009; Slowicka et al., 2020; Watanabe et al., 2003; Yang et al., 2013). One study, in which mice were treated with DSS describes increased neutrophil infiltration and colitis when macrophages and dendritic cells were depleted (Qualls et al., 2006). However, this study did not assess cancer endpoints. Overall, these findings reinforce a role for polarized macrophages as primary effector cells in the initiation of CRC.

In humans, the importance of MIBE and intestinal macrophages in CRC initiation has only been investigated indirectly. Bisphosphonates are a class of drugs that inhibit macrophage function (Rogers et al., 2011). They are primarily prescribed to treat or prevent high-risk postmenopausal osteoporosis (Tella and Gallagher, 2014) and act by inhibiting osteoclasts, the specialized multinucleated macrophages that resorb bone. Large, retrospective cohort studies of subjects taking bisphosphonates report that these drugs significantly reduce CRC risk (Bonovas et al., 2013; Thosani et al., 2013). Another line of evidence involves the analysis of tissue macrophages in human colon adenomas. These studies found macrophages in human colon adenomas are often polarized to an M1 phenotype and express PTGS2, iNOS and TNF-α (Ambs et al., 1998; Chapple et al., 2000; Hardwick et al., 2001; McLean et al., 2011). In the normal healthy colon, none of these enzymes are expressed. iNOS can be induced by multiple cells types, including polarized macrophages, and forms the radical nitric oxide that has competing effects on carcinogenesis (Janakiram and Rao, 2014). PTGS2, in particular, is a pro-carcinogenic enzyme that catalyzes the synthesis of prostaglandins with anti-apoptotic, pro-angiogenic, and pro-proliferative properties (Janakiram and Rao, 2014; Wu et al., 2010). Other less well-known by-products of M1-polarized macrophages include 4-HNE, a diffusible aldehyde and potent mutagen (Gueraud, 2017; Wang et al., 2012). Finally, both TNF-α and IL1β are secreted by M1-polarized macrophages. These cytokines activate multiple signaling pathways in colon epithelial cells that inhibit apoptosis, generate mutations and promote inflammation (Lasry et al., 2016; Rao et al., 2006; Yan et al., 2006; Yang et al., 2012).

### MIBE and induced pluripotency

MIBE represents a source of colon epithelial cell injury that can lead to reprogramming and induced pluripotency of fully differentiated post-mitotic cells. Under chronic mutagenic and proliferative pressures, these cells are primed to enter a pathway towards transformation (Wang et al., 2017). However, current evidence for MIBE remains limited. The repetitive *in vitro* exposure of primary murine colon epithelial cells to *E. faecalis*-triggered MIBE increased the expression of *Dclk1* and other stem-like markers. The end result was malignant transformation (Wang et al., 2015, 2017). Cancerous clones from these experiments showed the activation of Myc and the induction Klf4, Oct4 and Sox2 (Wang et al., 2017). These transcription factors are considered key regulators of induced pluripotency (Takahashi and Yamanaka, 2006).

Colonic epithelial cells that express *Dclk1* are attractive candidates for MIBE-induced pluripotency and as cells-of-origin for CSCs within CRCs (Chandrakesan et al., 2014; Nakanishi et al., 2013; Schwitalla et al., 2013; Westphalen et al., 2014). Intestinal epithelial cells that express this kinase participate in the repair of mucosal injury and DNA damage (Chandrakesan et al., 2016; May et al., 2014). In *Apc*^Min/+^ mice, Dclk1-positive colon epithelial cells show increased expression of pluripotency and self-renewal markers, and knockdown of *Dclk1* in these mice attenuated intestinal adenoma formation (Chandrakesan et al., 2017). Finally, the induction of pluripotency in post-mitotic colon epithelial cells through MIBE could be consistent with the top-down morphogenesis as seen in colon adenomas (Fig. 3). MIBE arises from the polarization of – otherwise tolerogenic – colon macrophages, followed by reprogramming and damage to bystander (or target) epithelial cells. These effects include genomic instability, induced pluripotency and malignant transformation. However, despite evidence that MIBE can be generated *in vitro* and *in vivo* by selected members of the gut microbiome, direct evidence for this process in human disease has not – yet – been obtained. MIBE mediators, such as TNF-α, 4-HNE and ROS, have been detected in human colon adenomas, although such associations do not prove causation and many questions remain unanswered. For example, does intestinal dysbiosis in humans promote the polarization of colon macrophages? Does human aging sensitize intestinal macrophages to pathogenic polarization by the gut microbiome? What evidence is there for induced pluripotency in dysplastic cells that form small human adenomas with top-down morphology? Are biochemical signatures for paligenosis or epigenetic change evident in these adenomas? And, finally, does MIBE generate unique mutational signatures in CRC? Answers to these questions and any evidence thereof would support MIBE as an important mechanism of carcinogenesis in human disease and would further advance our understanding of how the gut microbiota initiates sporadic CRC.

## Conclusions and future directions

Sporadic CRC arises within a complex milieu of overlapping risk factors that include age, host genetics, lifestyle and the gut microbiota. Understanding the mechanisms of microbiota-induced CRC initiation is essential for developing new prevention strategies for this disease. Recent investigations have identified several members of the human microbiota that express phenotypes that contribute to CRC initiation. The mechanisms by which human microbiota contribute to CRC initiation include the activation of β-catenin/Myc signaling, production of ROS, expression of DNA-damaging toxins and microbiota-induced polarization of colon macrophages to generate a bystander effect. Although there is limited evidence for MIBE contributing to CRC initiation in humans, the histology and immunology of sporadic human colon adenomas – together with findings from multiple animal models – link MIBE to parainflammation, paligenosis and top-down morphogenesis, as seen in precursor lesions to CRC. As such, MIBE has the potential to support multiple mechanisms by which gut bacteria initiate sporadic CRC. It provides a broad context for the major risk cofactors of CRC and helps to explain the preventive efficacy of anti-inflammatory drugs. Nevertheless, additional work is needed to better understand how intestinal barrier function, dysbiosis, and macrophage tolerance and responsiveness change with age. Finally, despite much recent progress, there are insufficient data on members of the gut microbiota that can chronically polarize colon macrophages and generate MIBE. Exploring these issues will help clarify how aging, intestinal dysbiosis and immunosenescence drive initiation of CRC.

In conclusion, a healthy and stable gut microbiota helps to maintain the mutually beneficial relationship between these microorganisms and the immune system. Perturbations of intestinal homeostasis due to aging and/or immunosenescence permits the emergence of bacterial pathobionts that can initiate DNA damage and reprogramming in colon epithelial cells through a bystander effect and, potentially, lead to malignant transformation over time. Unraveling the spectrum of mutations, epigenetic changes and induced pluripotency that occur in CRC is important in order to identify the cells-of-origin for CSCs within CRCs and to provide new strategies that can be used to prevent this disease.
